# A Stock Market Forecasting Model Combining Two-Directional Two-Dimensional Principal Component Analysis and Radial Basis Function Neural Network

**DOI:** 10.1371/journal.pone.0122385

**Published:** 2015-04-07

**Authors:** Zhiqiang Guo, Huaiqing Wang, Jie Yang, David J. Miller

**Affiliations:** 1 Key Laboratory of Fiber Optic Sensing Technology and Information Processing, School of Information Engineering, Wuhan University of Technology, Wuhan, China; 2 Department of Financial Math and Financial Engineering, South University of Science and Technology of China, ShenZhen, China; 3 Department of Electrical Engineering, The Pennsylvania State University, University Park, Pennsylvania, United States of America; University of Rijeka, CROATIA

## Abstract

In this paper, we propose and implement a hybrid model combining two-directional two-dimensional principal component analysis ((2D)^2^PCA) and a Radial Basis Function Neural Network (RBFNN) to forecast stock market behavior. First, 36 stock market technical variables are selected as the input features, and a sliding window is used to obtain the input data of the model. Next, (2D)^2^PCA is utilized to reduce the dimension of the data and extract its intrinsic features. Finally, an RBFNN accepts the data processed by (2D)^2^PCA to forecast the next day's stock price or movement. The proposed model is used on the Shanghai stock market index, and the experiments show that the model achieves a good level of fitness. The proposed model is then compared with one that uses the traditional dimension reduction method principal component analysis (PCA) and independent component analysis (ICA). The empirical results show that the proposed model outperforms the PCA-based model, as well as alternative models based on ICA and on the multilayer perceptron.

## Introduction

In recent years, it is an important issue in investment/financial decision-making and is currently receiving considerable attention from the research community [1]. The stock market is quite attractive if its behavior can be predicted; however, forecasting the stock market index is regarded as a difficult task due to its random walk characteristic. According to the Efficient Market Hypothesis [2], changes in stock market prices are determined by new information, but because the new information is unpredictable, the stock market price is also unpredictable. Some researchers argue that the stock market can be predicted over the short term, as reported in studies by Los [3] and Haugen [4]. China’s stock market is now the second largest in Asia following only Japan. Guo [5] indicated that the Chinese stock market has been gradually acting as the barometer of the economy since 2002. The Shanghai stock market opened in 1991, which plays an important role in Chinese economic development, so an increasing number of forecasting models are being developed to predict Shanghai stock market trends. These earlier studies have been reported in Cao et al. [6], Yang et al. [7], Zhang et al. [8], Dai et al.[9] and Ye et al. [10].

Over the past two decades, many models based on soft computing have been proposed [11–16]. In the most existing prediction approaches, there have been numerous studies using RBFNN for stock price prediction. RBFNN was first used to solve the interpolation problem of fitting a curve exactly through a set of points [17]. Versace et al. [18] used a mixture of RBFNNs to evaluate the performance of a heterogeneous mixture of neural network algorithms for predicting the exchange-traded fund DIA (AMEX ticker: DIA). Wang et al. [19] obtained the fractal dimension of the Shanghai stock market through a function approximation algorithm based on the RBFNN. Sun et al. [20] proposed a financial index forecasting model based on a modified RBFNN to find the important points of the stock index. A large number of successful applications have shown that RBFNN can be useful techniques for stock price forecasting due to their ability to approximate any continuous function with arbitrary precision. Since RBFNN has fast convergence, but a powerful nonlinear problem-solving ability. It motivates this study of utilizing RBFNNN for stock price prediction.

When using RBFNN for stock prices forecasting, the observed original values of prediction variables are usually directly used to build prediction models. One of the key problems is the inherent noise of original values affecting the prediction performance. Many studies on time series analysis have suggested that raw data preprocessing is useful and necessary for improving system performance and model generalization to unseen data. For stock market forecasting, as new data is obtained, if the predictive model can be refined to account for it, then the model should be better adapted for the new data, and its predictive accuracy should be improved. Thus, especially for predicting the stock market, with its inherent volatility, the predictive model should be dynamically learned on-line. In this learning context, the dimensionality of the raw data play an important role in improving the performance and reducing the computational complexity needed to learn the predictive model. In this case, many hybrid system methods were proposed to improve the performance of stock market forecasting systems [21–23]. These existing methods usually contain two stages, the first stage is feature extraction to remove the noise, the second stage is a predictor to forecast the stock price.

Atsalakis et al. [22] have pointed out that not all articles provide details of data preprocessing, or indeed whether any preprocesses are used. This indicates that more attention should be paid to the preprocessing methods used in stock market forecasting. In particular, more effective dimension reduction methods should be introduced to improve the performance of the forecasting model. Common approaches include data normalization, indicator reduction, and PCA [24], a very popular subspace analysis method which is successfully applied in many domains for dimension reduction. Ajithet et al. [23] used PCA to preprocess the raw data for stock market forecasting, but did not give details. Huang et al. [25] proposed a model based on PCA and BPNN to forecast the trends of the future’s price, and tested actual instances to validate that the performance of PCA+BPNN was preferable to that of a standard neural network. Ravi et al. [26] proposed and implemented a fusion model by combining PCA and RBFNN to build a bank performance prediction system. Tsai [27] use PCA as a feature selection method of stock prediction. Another well-known approach is ICA. Huang et al. [28] proposed a forecasting model that combined ICA and RBFNN to predict the trend in the real exchange rate. Huang [29], Guo [30] and Yeh [31] proposed a hybrid model by combining ICA and SVR in conducting time series prediction tasks. In these methods, PCA or ICA were used as preprocessing tool before building a stock prediction model.

PCA or ICA is suitable when the format of raw data is a vector with lower dimension. However, this condition is often not satisfied with the stock prediction. In multivariable prediction systems, there is a strong correlation between the variables, and the initial format of the raw data is a tensor. As feature extraction tools, both PCA and ICA need to transform the tensor into a vector, which contains two drawbacks. One is it requires prohibitive computational complexity, the other is PCA and ICA break the correlation residing in the raw data. In this study, a tensor subspace method, (2D)^2^PCA [32] based denoising scheme is proposed and integrated with RBFNN for building a stock price forecasting model (called (2D)^2^PCA+RBFNN model). In this work, first, a sliding window and 36 technique variables were used to obtain a multidimensional representation of the forecasting variable. Second, (2D)^2^PCA was applied to extract features from the predictor variables. Third, the features were used as the inputs of RBFNN. We attach importance to the influence of dimension reduction on the performance of the forecasting system. The proposed (2D)^2^PCA+RBFNN model use (2D)^2^PCA to remove the noise from the input raw data, the feature will contain less noise information and serve as the input of the RBFNN to predict the value or movement of the next day’s closing price. Compare with PCA and ICA, (2D)^2^PCA, as demonstrated in this paper, provides both computationally efficient preprocessing and more powerful feature extraction, leading to more accurate forecasting.

In previous studies, different stock markets have been modeled. Some scholars have focused on stocks, while others paid more attention to the stock market index, which represents the movement average of many individual stocks. Compared with a single stock, the stock market index remains relatively stable in reflecting overall market movement. The Shanghai stock market index collected from the China stock market is used to illustrate the proposed two-stage model. The prediction performance of the proposed approach is compared with other alternative approaches: the integration of PCA with RBFNN (called PCA+RBFNN), ICA with RBFNN (called ICA+RBFNN), PCA with BPNN (called PCA+BPNN) and ICA with BPNN (called ICA+BPNN). The model comparison shows that the proposed approach gets a better performance than the other alternative models.

The rest of this paper is structured as follows. In Section 2, we give a brief overview of (2D)^2^PCA and RBFNN. The proposed model is presented in Section 3. In Section 4, experiments are conducted to evaluate the performance of the proposed model. The conclusion is given in Section 5.

## Research Methodology

In this section, we briefly review the basic concepts about the underlying technologies used in the study.

### PCA

PCA is a well-known dimension reduction method used in pattern recognition and signal processing. Given *N* samples *A =* {*A*_1_,*A*_2_,*···*,*A*_*N*_} with the *i*-*th* sample *A*_*i*_ being an *m*×*n* matrix, one transforms *A*_*i*_ into a 1D vector *x*_*i*_ column by column or row by row, where *x*_*i*_ is an *mn*×1 column vector. The total scatter matrix of all samples is defined as follows:

$$C=\frac{1}{N}\sum_{i=1}^{N}\left(x_{i}-\bar{x}\right){\left(x_{i}-\bar{x}\right)}^{T}=XX^{T}$$ (1)

Here, $\bar{x}$ is the mean of *x*_*i*_. The principal vector of PCA is the eigenvector corresponding to the maximum eigenvalue of *C*. Generally, it is not enough to have only one optimal projection vector, so the discriminant vector *v*_*d*_ is composed of the orthogonal eigenvectors of *C* corresponding to the first *d* largest eigenvalues. The resulting feature vector for *x*_*i*_ is *y*_*i*_ obtained by projecting *x*_*i*_ into the subspace *v*_*d*_, i.e.

$$y_{i}=v_{d}x_{i},\begin{array}{cc} & i=1,2,\cdots ,N \end{array}$$ (2)

Setting a threshold *θ*,*d* can be selected as the smallest number of dimensions satisfying the following:

$$\frac{\sum_{i=1}^{d}\lambda_{i}}{\sum_{i=1}^{N}\lambda_{i}}\geq \theta$$ (3)

From the above, we can see that there are some disadvantages of PCA. First, the sample is transformed from a 2D to a long 1D vector, which breaks the spatial structure of the original matrix. Retaining this 2D structure may be critically important, when transforming the data to extract features. Second, and perhaps more importantly, due to the high dimension, it may be difficult to accurately evaluate the covariance matrix *C*, given a finite training set. Finally, the processing time taken by PCA may be prohibitive. To overcome these problems, Yang et al. [33] proposed Two-dimensional Principal Component Analysis (2DPCA) which was successfully used in face recognition.

### (2D)^2^PCA

Given the *N* sample matrix *A* *=* *{A*_1_,*A*_2_,*···*,*A*_*N*_}, *A*_*i*_ an *m×n* matrix, the covariance matrix can be defined as follows:

$$S_{t}=\frac{1}{N}\sum_{i=1}^{N}\left(A_{i}-\bar{A}\right){\left(A_{i}-\bar{A}\right)}^{T}$$ (4)

In (Eq 4), $\bar{A}=\frac{1}{N}\sum_{i=1}^{N}A_{i}$ is the mean of all samples. One can compute the eigenvalue *λ*_*i*_ and eigenvector *v*_*i*_ of *S*_*t*_. Accordingly the projecting subspace *V*_*d*_ is composed of the orthogonal eigenvectors *v*_1_,*v*_2_,*···*,*v*_*d*_ of *S*_*t*_ corresponding to the first *d* largest eigenvalues. The feature matrix of 2DPCA is $y_{i}=A_{i}^{T}V_{d}$ obtained by projecting *A*_*i*_ into the subspace *V*_*d*_ and the size of *y*_*i*_ is *n×d*.

In the 2DPCA algorithm, the size of the covariance matrix *S*_*t*_ is *m×m*, much smaller than that of PCA. For this reason, the computational complexity of 2DPCA is far less than that of PCA. At the same time, because the covariance matrix is built up by *A*_*i*_, the information of the spatial structure is retained in the processing. However, the main disadvantage of 2DPCA is that the feature values are often much larger than those of PCA. Furthermore, some studies indicate that 2DPCA is essentially working in the column direction of the matrix, i.e., it extracts the features of the matrix only in the column direction; the information in the column direction is uncorrelated, but the information in the row direction is still correlated after the transformation. Zhang et al (2005) proposed a Two-directional Two-dimensional method called (2D)^2^PCA to address this problem.

Suppose the projection matrix *V*_*d*_ has been obtained. Project the *m×n* matrix *A*_*i*_ into *V*_*d*_ to yield an *n×d* feature matrix$y_{i}=A_{i}^{T}V_{d}$. Then *y*_*i*_ is transposed to yield$y_{i}^{T}$. After that, regard $y_{i}^{T}$ as the new training sample on which to carry out 2DPCA again (called alternatve 2DPCA), yielding the feature matrix *z*_*i*_, If *p* eigenvectors are selected in alternative 2DPCA to form the projecting subspace *W*_*p*_, the (2D)^2^PCA algorithm can be described as follows:

$$z_{i}=V_{d}^{T}A_{i}^{}W_{p}$$ (5)

The size of *z*_*i*_ is *d×p*, since *d<<m* and *p<<n*, the dimension of this matrix is reduced significantly, compared with alternative transformations. From Formula (5) we can see that the main idea of (2D)^2^PCA is that the original matrix *A*_*i*_ is projected into a 2DPCA subspace to extract the row direction feature *y*_*i*_, then transposed to yield$y_{i}^{T}$, and the alternative 2DPCA is utilized to extract the column direction feature. Thus, the feature matrix *z*_*i*_ contains both the row direction and the column direction feature information from the original matrix. The feature set obtained from 2DPCA is generally much higher dimensional than that of (2D)^2^PCA. So from the standpoint of dimension reduction, the performance of 2DPCA is much worse than that of (2D)^2^PCA. For on-line stock forecasting systems, if 2DPCA (rather than (2D)^2^PCA) is used as a tool to preprocess raw data, then the training complexity of the model will be drastically increased. For more details please refer to [32].

### RBFNN

**Fig 1:**
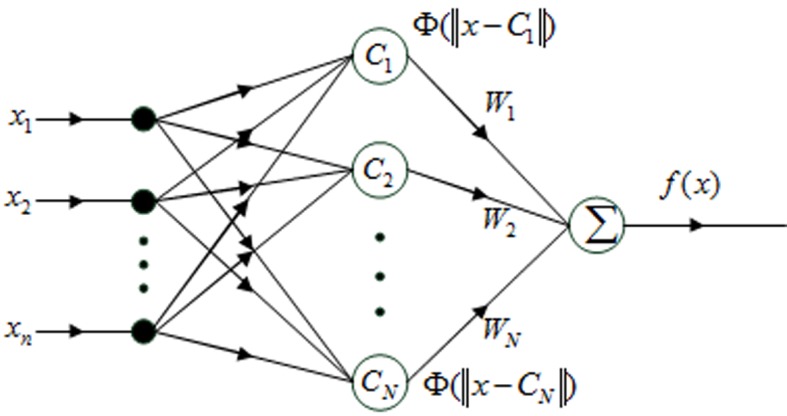
The architecture of RBFNN. *x =* [*x*_1_,*x*_2_,*···*,*x*_*n*_] is n-dimensional input vector, *C*_*i*_(*i =* 1,2,*···*,*N*) is the center of transformation function Φ(*x*), *W* = [*W*_1_,*W*_2,_ *···*,*W*_*N*_] is the weight between the hidden layer and output layer.

The idea of RBFNN [30] derives from the theory of function approximation. The RBFNN has a three-layered structure: the input layer, the hidden layer and the output layer. The input layer collects and feeds the input data to each node of the hidden layer. The hidden nodes implement a set of radial basis functions which are often chosen to be Gaussian functions. The output layer implements a weighted linear summation function to sum the outputs of the hidden layer to yield the prediction value (which may be thresholded, if a binary decision is sought). The RBFNN architecture is shown in Fig 1.

In Fig 1, *x* and *f*(*x*) are the input and output of the RBFNN network respectively. *W*_*i*_ is the output weight between hidden unit *i* and the output unit. Φ(*x*) is the transformation function of the hidden layer, which is defined by activation functions with a local field of response at the output. When the Gaussian function is used, the common form of *f*(*x*) for an RBFNN is as follows:

$$f(x)=\sum_{i=1}^{N}W_{i}\exp\left(-\frac{\left\|x-c_{i}\right\|}{2\sigma^{2}}\right)$$ (6)

Here, σ is the width of the Gaussian, *c*_*i*_ is the data center of the basis function. The network training is divided into two steps: first the weights from the input to the hidden layer are determined; then the weights from the hidden layer to the output layer.

## Proposed Forecasting Model

### The input and output of the system

In this study, we have two goals: one is to predict the *t*+1 day’s closing price by using the *N* days’ data preceding day *t*+1, and the other is to demonstrate the accuracy of the model in predicting the stock price movement. The Shanghai stock index is used for testing the proposed method and comparing with the PCA and ICA dimension reduction approach.

There are two important factors regarding the input data. The first concerns the variables from the price history for each day. In previous studies, many technical variables have been proposed as the features to predict the trend of the stock market, such as closing price, moving average line, Williams index and so on. Different models apply different variables and there is no unified framework for the selection of input variables. For example, Teixeira et al [34] and Ettes [12] selected only two input variables. This is quite different from Zorin et al. [35] who used 61 variables. For our study, we believe too few variables will fail to represent the intrinsic features of the stock market, and too many variables will lead to computational (and potentially model generalization) difficulties. In [36], 22 variables were selected as input to the prediction model and satisfactory results were achieved. As the stock market price is determined by various economic and non-economic factors, it is difficult to predict stock market trends using only a few factors. For this reason, with reference to [34] and [37], we selected 36 variables for each day as the input to the prediction model, as reported in Table 1. *x*(*t*),*x*_*h*_(*t*),*x*_*l*_(*t*) *and x*_*o*_(*t*) mean the stock’s close, high, low and open prices, respectively, on day *t*. Other variables are calculated based on *x*(*t*),*x*_*h*_(*t*),*x*_*l*_(*t*) *and x*_*o*_(*t*), and the description and formulae of the variables are displayed in Table 1. The principle that we used to select the variables is three folds: The first part is the basic data of the stock market, which can be directly obtained from the stock market database. These variables include *I*_1_ to *I*_4_. The second part is technical variables which are commonly used by some investors. For example, Moving average, Williams index, and so on. These variables include *I*_5_ to *I*_25._ The third part is the movement of basic data or technical variables, which represent the trend of changes in the data. These variables include *I*_26_ to *I*_36_.

**Table 1:** Variables used as inputs.

**name**	**Description or Formula**
*I*_*1*_ *= x*_*o*_(*t*)	Open price
*I*_*2*_ *= x*_*h*_(*t*)	High price
*I*_*3*_ *= x*_*l*_(*t*)	Low price
*I*_*4*_ *= x*(*t*)	Close price
*I*_*5*_ *= MA*5 *I*_*5*_ *= MA*10 *I*_*7*_ *= MA*20	Moving average
*I*_*8*_ *= BIAS*5 *I*_*9*_ *= BIAS*10	BIAS
*I*_*10*_ *= DIF*	EMA12-EMA26
*I*_*11*_ *= DEA*5 *I*_*12*_ *= DEA*10	Difference and Equal average
*I*_*13*_ *= K I*_*14*_ *= D*	Stochastic %K %D
*I*_*15*_ *= ROC*	Price rate of change
*I*_*16*_ *= TR*	True range of price Movements
*I*_*17*_ *= MTM*6 *I*_*18*_ *= MTM*12	Momentum
*I*_*19*_ *= WR%*10 *I*_*20*_ *= WR*%5	Williams index
*I*_*21*_ *= OSC*6 *I*_*22*_ *= OSC*12	Oscillator
*I*_*23*_ *= RSI*6 *I*_*24*_ *= RSI*12	Relative strength index
*I*_*25*_ *= PSY*	Phycholoigical Line
*I*_*26*_	*K*(*t*)-*K*(*t-1*)
*I*_*27*_	*D*(*t*)-*D*(*t-1*)
*I*_*28*_	(*x*(*t*)-*x*(*t-1*))/*x*(*t-1*)
*I*_*29*_	(*x*(*t*)-*x*_*o*_(*t*))/*x*_*o*_(*t*)
*I*_*30*_	(*x*(*t*)-*x*_*l*_(*t*))/(*x*_*h*_(*t*)-*x*_*l*_(*t*))
*I*_*31*_	(*MA*5(*t*)-*MA*5(*t-*1))/*MA*5(*t-*1)
*I*_*32*_	(*MA*20(*t*)-*MA*20(*t-*1))/*MA*20(*t-*1)
*I*_*33*_	(*MA*5(*t*)-*MA*20(*t-*1))/*MA*20(*t-*1)
*I*_*34*_	(*x*(*t*)-*MA*20(*t*))/*MA*20(*t*) (*x*(*t*)-min(*x*(*t*-1),*x*(*t*-2),…,(*t*-*N*)))/ min(*x*(*t*),*x*(*t*-1),…,(*t*-*N*))
*I*_*35*_	(*x*(*t*)-min(*x*(*t*-1),*x*(*t*-2),…,(*t*-*N*)))/min(*x*(*t*),*x*(*t*-1),*…*,(*t*-*N*))
*I*_*36*_	(*x*(*t*)-max(*x*(*t*-1),*x*(*t*-2),…,(*t*-*N*)))/max(*x*(*t*),*x*(*t*-1),*…*,(*t*-*N*))

*I*_*1*_ to *I*_*36*_ 36 variables are selected as the inputs of the forecasting model. The name and description of the variables are shown in the 1^st^ column and the 2^nd^ column, respectively.

The second key factor of the input data is the length of the sliding window. There lies in the fact that the direction of stock market indexes changes over the long run, and the price of the stock market is the result of “momentum” accumulated over a period of time. Thus, it is not reasonable to select data from only one day or several days to predict the next day's price. Some studies have also indicated that the near daily data had a bigger influence over the future price than data furthered in the past. In this study, 20 days are selected as the length of the sliding window for each variable. The input data include all 36 technical variables, each variable having 20 days of observation, which gives a 36×20 matrix. For example, if the prediction time is *t*+1, the input data are *I*(*t*-*j*,*i*)(*i* = 1,2,*…*,36;*j* = 0,1,2,*…*,19).

Different from the previous study, both historical data and the related technical variables are taken into the count in this approach. The raw input data is a natural tensor containing the correlation between technical variables. So it is important retaining this tensor structure on the feature extraction step. Obviously, it is difficult for conventional forecasting models to accept this huge data as input, thus the first step is to reduce the dimension of the raw data before sending it to the forecasting model.

In this study, we provide two kinds of output of the forecasting system. One is the prediction of the closing price *x*(*t*+1) on day *t*+1; the other is the prediction of the trend of closing price, which can be defined as follows:

$$y(t)=\{\begin{array}{cc} 1 & x\left(t+1\right)>x(t)\\ 0 & x(t+1)=x(t)\\ -1 & x(t+1)<x(t) \end{array}$$ (7)

### The overall framework of the model

**Fig 2:**
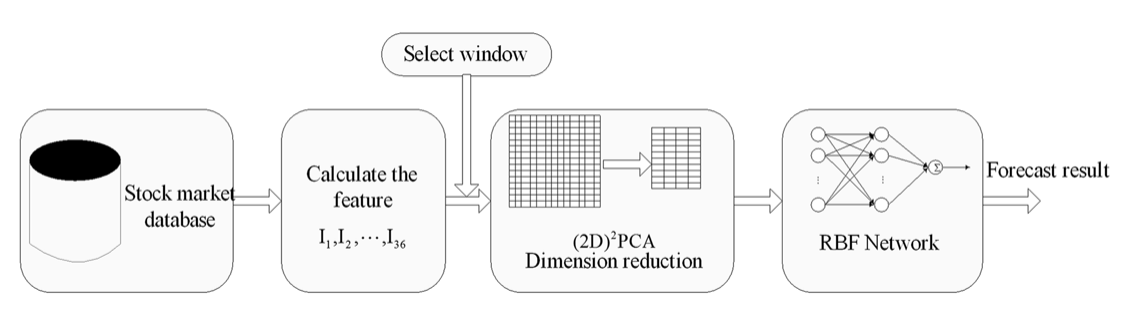
Diagram of (2D)^2^PCA+RBFNN forecasting model. The model is divided five modules, including the database of stock market, variables calculated module, sliding window, dimension reduction module and RBFNN predictor.

To predict future trends of a stock price, the system can be built up with the following four components: (1) the initial exploration, (2) calculation of variables, (3) dimension reduction using (2D)^2^PCA^2^, and (4) forecasting by the RBF network. Therefore, a research forecasting model (Fig 2) is presented to evaluate the performance of the proposed model. The data processing begins with the selection of data from the stock market database and ends with the prediction result of the closing price or movement of the closing price.

#### Input variables of the model

In the first module of the system, historical stock market data are selected from the database, such as the stock’s close, high, low and open price. After the raw data are prepared, they are sent to the second module of the system to compute the variables used as the raw features of the forecasting model. Based on Table 1, the first four variables *I*_1_ to *I*_4_ are the raw data, *x*_*o*_(*t*),*x*_*h*_(*t*),*x*_*l*_(*t*) and *x*(*t*), with the last 32 *I*_5_ to *I*_36_ technical variables calculated based on the given formulae.

#### Data collecting

**Fig 3:**
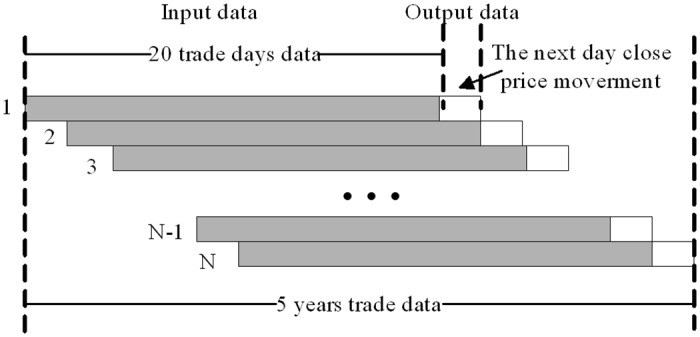
Diagram of building up the dataset. Stock time series segmentation is made by 20 width sliding window. The gray block represents the input data which including 20 trading day’s data. The white block represents the target output data which is the next day's closing price.

A sliding window is applied to the entire data set to extract the input raw data used by the forecasting model. As we have mentioned in 3.1, 20 is chosen as the length of the sliding window; as the window is moved from the beginning to the end of the data set, the training samples and testing samples are obtained sequentially. This process can be described as showed in Fig 3. The gray block represents the input data of the forecasting model which includes 20 trading days' data for the 36 technical variables. As the window is sliding, *N* input data are obtained from the trading data set. The data instances 1 to *N*_*i*_ are used as the training set, and the data from *N*_*i*_ *+*1 to *N* are used as the test set. The white block represents the target output data, which is the next day's closing price or the price movement. Corresponding to the input, targets 1 to *N*_*i*_ are used as the training targets, and targets *N*_*i*_ *+*1 to *N* are the test targets. A similar method was also reported in [34].

#### Dimension reduction

The function of the third module aims to reduce the dimension of the input data. Dimension reduction is a key step in signal processing and pattern recognition systems. It aims to filter out the information redundancy and extract the intrinsic features from high dimensional data. In this study, 36 technical variables were selected, and each is measured for 20 previous trading days; we note that the dimensionality of the input data is high, and the data likely will generally have some redundancy. In order to decrease computational complexity both of the system design and of the forecasting, it is both acceptable (from an information loss standpoint) and necessary to reduce the data dimensionality. We propose to use (2D)^2^PCA to extract features from the original data. An *m*×*n* matrix *A*_*i*_ is projected into the (2D)^2^PCA subspace to yield a *d*×*p* feature matrix *z*_*i*_,*d* and *p* can be selected based on (Eq 3). As discussed in previous section, the size of z_i_ is much smaller than that of *A*_*i*_, and thus *z*_*i*_ is chosen to be the input of the RBFNN.

#### Forecasting process

The last module of the system is the RBFNN, which accepts *z*_*i*_ from the (2D)^2^ PCA dimension reduction module and forecasts the next day's price or the price movement. The training set is used to learn the weights *W* ^*1*^ and *W* ^2^of the RBFNN. After the training is completed, the test set is used to evaluate the performance of the forecasting model. The input variables are not usually within the range [0 1] in the training set after dimension reduction; each data point is thus scaled to be within this range by (Eq 8):

$$z_{ij}=\frac{z_{ij}-\min(z_{i})}{\max(z_{i})-\min(z_{i})}$$ (8)

where *z*_*ij*_ is the *j-th* element of *z*_*i*_, min(*z*_*i*_) is the Minimum value of *z*_*i*_, and max(*z*_*i*_) is the Maximum value of *z*_*i*_.

The architecture of the RBFNN is as follows: the nodes of the input layer are equal to the data dimension reduced by PCA or (2D)^2^PCA; the output layer has 1 node. The first layer has Radial basis transfer function (RABAS) neurons, and calculates its weighted input with the Euclidean distance weight function, and calculates a layer's net input by combining its weighted inputs. The second layer has Linear transfer function (PURELINE) neurons, and calculates its weighted input with the Dot product weight function, and its net input with sum net input function. The training method used for the RBFNN is the Least Squares (LS) algorithm [38].

The following steps are repeated until the network's mean squared error falls below the GOAL or the maximum number of neurons are reached: ①The network outputs are generated for the training set; ② The input vector with the greatest error is found; ③A RADBAS neuron is added with weights equal to that vector; ④ The PURELIN layer weights are redesigned to minimize the training set mean-squared prediction error.

As discussed in Section RBFNN, transformation function Φ(*r*) of the hidden layer has to be determined when the RBFNN forecasting model is developed. In this work, Gaussian function is adopted because it is the most widely used transformation function and has performed well in most forecasting cases [39]. The performance of RBFNN also depends on the choice of the parameter σ (the width of the Gaussian). There are no general methods for setting σ. Selection is usually based on the cross-validation method or the user’s prior knowledge and expertise [40]. In this study, the grid search method [41] using exponentially growing sequences of σ is applied to identify good parameter. σ is selected through repeated and numerous experiments according to performance considerations.

### Analysis of the model

In this section, we give an intuitive analysis for the (2D)^2^PCA+RBFNN model. The performance advantage of the proposed model (which will be experimentally investigated in the next section) may lie on the following reasons.

First, traditional forecasting models may be classified as auto-regressive and multi-variable models. The former is based on the idea that all the related factors can be reflected in the closing price of the stock, so the closing price history decides the future trend. On the other hand, the latter model holds that some technical variables are very useful for making predictions, such as Moving average, Relative strength index, Oscillator, Williams index and so on. In our proposed model, the input data of the model is a matrix, with columns representing the technical variables and, rows representing the historical data for the technical variables, so the influence on the stock market price from both the technical variables and the historical data are taken into account.

Second, from the formulae in Table 1, we can see that the technical variables are correlated with each other. It is obvious that historical data within the sliding window are also correlated. So the input data have correlation both in the row direction and in the column direction. In the proposed model, (2D)^2^PCA is carried out to reduce the dimension of the raw data. The advantage of (2D)^2^PCA is its extraction of useful information by removing the correlation from both the row direction and the column direction. This fact suggests the potential improvement in performance, compared to models that do not decorrelate in both directions. The other key issue is the algorithm complexity of the methods. If the size of the training sample is *m*×*n*, let *d*_1_ and *d*_2_ be the number of row-projected and the column-projected vectors, respectively. The training complexity of (2D)^2^PCA and PCA are *O*(*n* ^*2*^ *d*_*1*_ *×m* ^2^ *d*_*2*_) and *O*(*n* ^*2*^ *×m* ^2^) respectively. Since *d*_1_ *<<n* and *d*_2_ *<<m*, the complexity of (2D)^2^PCA is much less than that of PCA. With respect to ICA, it is common to use PCA to whiten the raw data before ICA is calculated. So the complexity of ICA is much bigger than PCA. In this case, compared to ICA and PCA, (2D)^2^PCA accelerates the computational speed of forecasting by more efficient calculation.

Last but not least, RBFNN is used as the predictor. Compared to traditional neural networks, RBFNN has several distinct characteristics [42]. Firstly, it has the best approximation characteristic and no local minimum problem. Second, it has a strong robust and adaptive capability which can help it to give better forecasting results. Furthermore, it has fast convergence speed and good stability. For these reasons, RBFNN is widely used in pattern recognition and time series prediction. Since stock market data has random walk characteristics, stock market forecasting is a nonlinear regression problem. The characteristics of RBFNN are quite suitable to deal with such problems.

## Experimental Results and Analysis

### Data preparation

**Fig 4:**
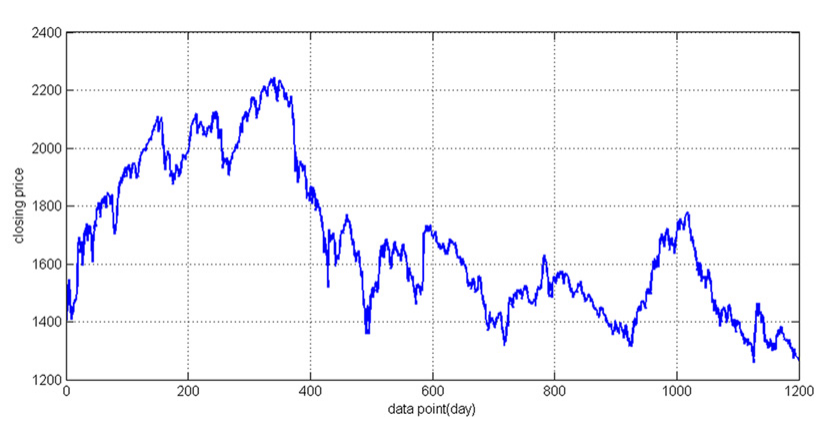
The closing price of raw data. Shanghai stock market index collected from 4 Jan. 2000 to 31 Dec. 2004 includes 1200 trading days' data.

In order to verify the effectiveness of the proposed model for forecasting, the Shanghai stock market index collected from 4 Jan. 2000 to 31 Dec. 2004 was used in this experiment. The overall data include 1200 trading days' data which are split into two parts: 4 Jan. 2000 to 31 Dec. 2003 and 1 Jan. 2004 to 31 Dec. 2004. The former, which includes 957 trading days' data, is used as the training set, and the latter, which includes 243 trading days' data, is used as the test set. The daily Shanghai stock market index closing prices are shown in Fig 4. As discussed in the last section, a sliding window is employed to build up the raw data of the training set, with 937 training samples obtained from 957 days of trading data. Each input training sample is a 36×20 matrix, the rows representing the technical variables shown in Table 1, and the column representing the past 20 days’ data for each technical variable. The target output of a training sample is the closing price for the next day. Experiments are performed on a PC with 2.30GHz PCU, 2G RAM memory, on a MATLAB 7.1(R2010a) platform.

### Experiment design

There are two purposes in this experiment. One is to test the validity of the proposed model for one-day-ahead forecasting of the stock price; the other is to compare the performance of the (2D)^2^PCA+RBFNN model with other related models, PCA+RBFNN model, ICA+RBFNN and PCA+BPNN model [26, 27, 29]. In the PCA models, the dimension of the input data is determined by (Eq 3). Experiments show forecasting results vary with the feature dimensionality; in this experiment, three scales are selected corresponding to *θ*. When *θ* is 0.99, 0.999 and 0.9999, the dimension of the PCA model and ICA model are 7, 50 and 128 respectively. Another experiment is conducted to compare the performance of the (2D)^2^PCA+BPNN with PCA +BPNN and ICA+BPNN models. Because the time required to train the BPNN in the case of high dimension is prohibitively high, the performance of the three models is only compared in the case of *Dim* = 7 in this experiment.

Here, the fixed-point algorithm [36] is carried out to implement ICA, and a method based on amplitude of the weight vector is used to determine the selection of the ICA subspace [43]. The training parameters for the RBFNN are: Mean squared error goal is 0; *SPREAD* is selected through repeated and numerous experiments according to performance considerations. It is found that the best *SPREAD* = 5×10^3^ for the range of *SPREAD* = *m*×10^n^(*m* = 1,2,*…*,9;*n* = 1,2,*…*,9).The maximum number of neurons was set equal to the number of training samples. In our experiment, the architecture of BPNN was chosen to be 7-10-1; that is, the input layer has 7 nodes, the hidden layer has 10 nodes and the output layer has 1 node. The hidden nodes were determined through trial and error because the BPNN does not have a general rule for selecting the optimal number of hidden nodes. The number of hidden layer and output layer transform functions were chosen to be the Hyperbolic tangent sigmoid transfer function and Linear transfer function respectively. The maximum training step epoch was 10000, the training error goal was 0.0009, the learning rate was 0.01, and the Additional Momentum Method was used to train the network. In order to compare with the PCA model, 7×1 = 7,7×7 = 49, and 11×11 = 121 were selected as the dimensions of the (2D)^2^PCA models. Clearly, the dimension of the (2D)^2^PCA model is equal to or even smaller than the PCA model and ICA model under the three different conditions.

To measure the performance of the proposed model, 12 performance indicators were selected. The descriptions and formulae of these indicators are described in Table 2. In these indicators, PCD, R^2^, *r*_1_, *r*_2_, MAPE, HR, TR, RMSE and SMAPE are used to measure whether the predicted value is similar to the actual value. If PCD, R^2^ and *r*_1_ are big, it means that the predicted result is similar to the actual value. If MAPE, RMSE and SMAPE are small, this also indicates that the predicted result is close to the actual value. HR is used to measure the prediction accuracy of the stock market trend. TR and *r*_2_ are applied to evaluate the return of different models. ET, TT and ST are used to test the effective computation time of the proposed model; the total running time of the proposed model is the sum of ET, TT and ST.

**Table 2:** Measure indicators.

**name**	**Description**	**Formula**
*r*_1_	correlation coefficient between actual value and prediction value	$r=\frac{\sum_{t=1}^{N}\left(y(t)-\bar{y}(t)\right)(\overset{⌢}{y}(t)-\bar{\overset{⌢}{y}}(t))}{\sqrt{\sum_{t=1}^{N}{\left(y(t)-\bar{y}(t)\right)}^{2}{\left(\overset{⌢}{y}(t)-\bar{\overset{⌢}{y}}(t)\right)}^{2}}}$
*R* ^2^	Non-linear regression multiple correlation coefficient	$R^{2}=1-\frac{\sum_{t=1}^{N}{\left(y(t+1)-\overset{⌢}{y}(t)\right)}^{2}}{\sum_{t=1}^{N}{\left(y(t)-\bar{y}(t)\right)}^{2}}$
*r*_2_	correlation coefficient between actual return and prediction return	$r=\frac{\sum_{t=1}^{N}\left(r_{e}(t)-{\bar{r}}_{e}(t)\right)({\overset{⌢}{r}}_{e}(t)-{\bar{\overset{⌢}{r}}}_{e}(t))}{\sqrt{\sum_{t=1}^{N}{\left(r_{e}(t)-{\bar{r}}_{e}(t)\right)}^{2}{\left({\overset{⌢}{r}}_{e}(t)-{\bar{\overset{⌢}{r}}}_{e}(t)\right)}^{2}}}$
*PCD*	Percentage of correct direction	$\begin{array}{l} PCD=\frac{1}{N}\sum_{t=1}^{N}Pcd_{t}\\ Pcd_{t}=\{\begin{array}{cc} 1 & \left(y(t+1)-\overset{⌢}{y}(t)\right)\left(y(t+1)-y(t)\right)>0\\ 0 & else \end{array} \end{array}$
***SMAPE***	Symmetric mean absolute percentage error	$MAE=\frac{1}{N}\sum_{t=1}^{N}2\left|y(t)-\overset{⌢}{y}(t)\right|/\left|y(t)+\overset{⌢}{y}(t)\right|$
***MAPE***	Mean Absolute Percentage Error	$MAPE=\frac{1}{N}\sum_{t=1}^{N}\left|y(t)-\overset{⌢}{y}(t)\right|/y(t)$
***RMSE***	Root mean square error	$RMSE=\sqrt{\frac{1}{N}\sum_{t=1}^{N}{\left(y(t)-\overset{⌢}{y}(t)\right)}^{2}}$
***HR***	Hit rate	*HR = N_p_/N*
***TR***	Total Return	$TR=\sum_{t=1}^{N}\left[y(t+1)-y(t)\right],if\begin{array}{cc} \overset{⌢}{y}(t+1)>y(t) & \end{array}$
***ET(s)***	Extracting feature time	—
***TT(s)***	Training time	—
***ST(s)***	Simulation time	—

11 variables are selected as the measurement of the foresting model. The name, description and formula are displayed in the 1^st^ column, 2^nd^ column and 3^rd^ column, respectively.

### Results and discussion

**Fig 5:**
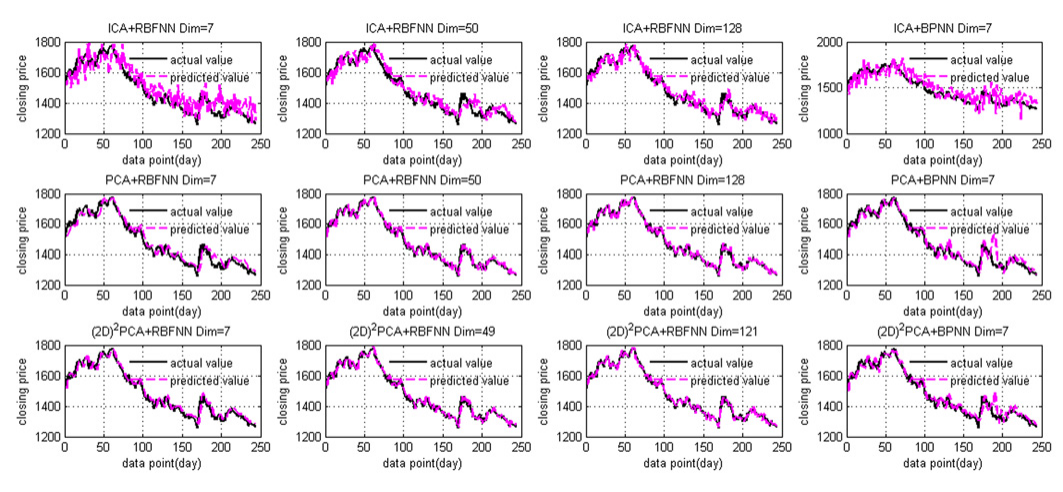
Fitting curve of prediction results and the actual data. The red and black colored curves indicate the prediction results and actual data, respectively. The top, middle and bottom row display the prediction results of ICA, PCA and (2D)^2^PCA associated with RBFNN (Dim = 7,50,128) and BPNN (Dim = 7), respectively.

**Fig 6:**
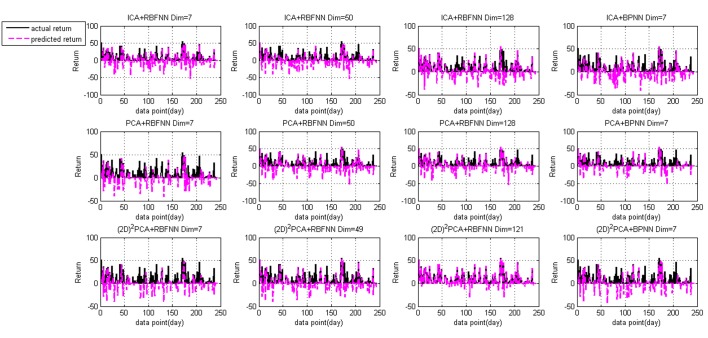
Curve of return time series. The red and black colored curves indicate the prediction return and actual return, respectively. The top, middle and bottom row display the returns of ICA, PCA and (2D)^2^PCA associated with RBFNN (Dim = 7,50,128) and BPNN (Dim = 7), respectively.

**Fig 7:**
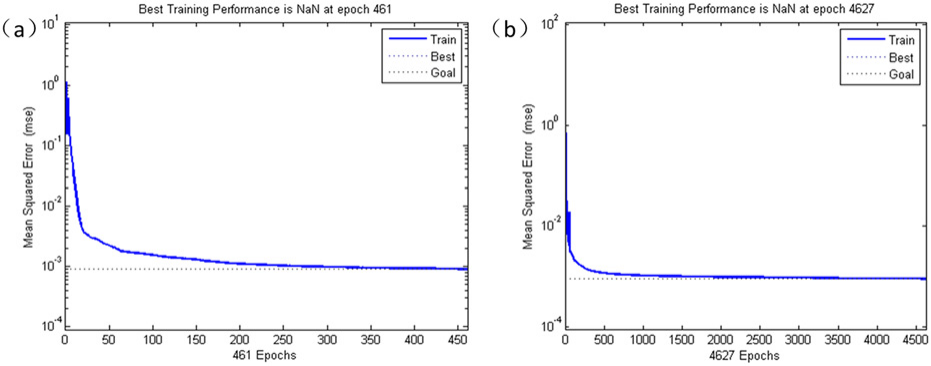
The training process of the BP network. (a)The (2D)^2^PCA+BPNN (DIM = 7×1 = 7) model converge after 461 epochs. (b)PCA+BPNN (DIM = 7) converges after 4627 epochs.

The experimental results are depicted in Figs 5–7 and S1 Data. Tables 3 and 4 summarize the empirical results of the proposed model, PCA+RBFNN, ICA+RBFNN, (2D)^2^PCA+BPNN, ICA+BPNN and PCA+BPNN models on stock price forecasting. Fig 5 presents the fitting curve of prediction values and actual values, Fig 6 displays the return under four conditions. In the Fig 5, the blue curve represents the actual data and the red curve represents the prediction. The bottom line of Figs 5 and 6 shows the fitness curves of proposed model and (2D)^2^PCA+BPNN model; the middle line shows the fitness curves using the PCA+RBFNN and PCA+BPNN model; and the top line Fig 5 shows the fitness curves using the ICA+RBFNN and ICA+BP model.

**Table 3:** Nine measure indicators of ICA, PCA and (2D)^2^PCA associated with RBFNN and BPNN under different dimension.

Group	Method	*DIM*	*PCD*	*R* ^*2*^	*TR*	*MAPEr*_*1*_	*r*_*1*_	*r*_*2*_	*RMSE*	*SMAPE*	*HR*
1	(2D)^2^PCA+RBFNN	11×11 = 121	0.75309	0.99369	**1317.7**	6.6305e-04	0.9969	0.9458	12.135	0.0066354	0.73251
	PCA+RBFNN	128(E>0.9999)	0.5679	0.98493	242.7	9.7919e-04	0.9927	0.6806	18.525	0.0097724	0.64609
	ICA+RBFNN	128	0.5638	0.9555	352.2	3.3e-03	0.9800	0.6972	30.840	0.0186000	0.57610
2	(2D)^2^PCA+RBFNN	7×7 = 49	0.51852	0.9854	93.1	0.0022000	0.9927	0.6535	18.425	0.0097841	0.5679
	PCA+RBFNN	50(E>0.999)	0.4737	0.9833	-20.9	0.0103340	0.9918	0.6593	19.599	0.010327	0.54773
	ICA+RBFNN	50	0.5432	0.9253	-89.6	0.0025000	0.9668	0.5959	40.170	0.034800	0.55560
3	(2D)^2^PCA+RBFNN	7×1 = 7	0.49794	0.96573	-255.7	0.0019000	0.9853	0.4952	27.033	0.014945	0.53086
	PCA+RBFNN	7(E>0.99)	0.48971	0.9642	-56.2	0.0153700	0.9757	0.5338	29.571	0.015367	0.51440
	ICA+RBFNN	7	0.4938	0.7307	26.8	0.0091530	0.9103	0.6742	67.170	0.066900	0.49790
4	(2D)^2^PCA+BPNN	7×1 = 7	0.5026	0.95073	-72.6	8.189e-4	0.9779	0.5744	29.215	0.016477	0.50617
	PCA+BPNN	7(E>0.99)	0.49794	0.93699	-14.8	0.0202100	0.9678	0.6215	35.819	0.020097	0.49383
	ICA+BPNN	7	0.4650	0.6927	-143.6	0.0026000	0.8678	0.5716	80.510	0.084100	0.47740
5	raw data+RBFNN	36×20 = 720	0.6914	0.9687	948.3	0.0041	0.9884	0.8410	25.090	0.013600	0.69420

The predictor in group 1,2,3 and 5 is RBFNN, and the predictor in group 4 is BPNN.

**Table 4:** The running time of ICA, PCA and (2D)^2^PCA associated with RBFNN and BPNN under different dimension.

**Group**	**Method**	**DIM**	**ET(s)**	**TT(s)**	**ST(s)**	**Total Time(s)**
1	(2D)^2^PCA+RBFNN	11×11 = 121	0.369967	2.170977	0.484439	3.025383
	PCA+RBFNN	128(E>0.9999)	9.491866	3.343269	0.494813	13.330248
	ICA+RBFNN	128	17.096262	3.456170	0.470561	21.022993
2	(2D)^2^PCA+RBFNN	7×7 = 49	0.182625	0.977762	0.154389	1.314776
	PCA+RBFNN	50(E>0.999)	7.518724	1.233592	0.169199	8.921515
	ICA+RBFNN	50	14.037029	1.314493	0.115149	15.466671
3	(2D)^2^PCA+RBFNN	7×1 = 7	0.128442	0.734274	0.086912	0.969428
	PCA+RBFNN	7(E>0.99)	6.962491	0.932658	0.028345	7.923494
	ICA+RBFNN	7	14.366689	1.117943	0.028196	15.512828
4	(2D)^2^PCA+BPNN	7×1 = 7	0.114934	19.034696	0.040818	19.190448
	PCA+BPNN	7(E>0.99)	6.996199	26.816377	0.045035	33.857611
	ICA+BPNN	7	14.028339	85.574272	0.042313	99.644924
5	raw data+RBFNN	36×20 = 720	0	8.942768	4.769554	13.712322

The predictor in Group 1,2,3 and 5 is RBFNN, and the predictor in Group 4 is BPNN.

From Fig 5, we can see that the prediction results of the proposed model are much closer to the actual data than the other models. Comparing PCA and ICA models, it is clear that the (2D)^2^PCA+RBFNN model shows better performance. The performance of the three models changes with the dimension of the input data—the prediction performance improves with increasing dimension.

Observing the graphs of returns of Fig 6, different models are tried as predictor based on return to find out which model gives out the best result. In the Fig 6, if the point of return is above zero, it means the return is positive and the investor can profit. More point above zero, better performance the model has. From the result, we can see (2D)^2^PCA+RBFNN model obtains the best result with the dimension being 121 since most of the points are above zero. The total returns of different models are listed in Table 3.

Fig 7 shows the training process. The (2D)^2^PCA+BPNN model converges after 461 epochs, while PCA+BPNN converges after 4627 epochs which is much slower convergence than the (2D)^2^PCA+BPNN model. For ICA+BPNN, it is hard to meet convergence—the training steps reached the maximum of 10000. The results show that, for BPNN, the data processed by (2D)^2^PCA has better convergence performance than that of PCA and ICA.

Table 3 and Table 4 compare the forecasting results of the (2D)^2^PCA model with the PCA model, ICA model and raw data model. The results indicate that the (2D)^2^PCA+RBFNN model outperforms the PCA+RBFNN model and; that the (2D)^2^PCA +BPNN model outperforms both the PCA+BPNN model and the ICA+BPNN model. At the same time, the (2D)^2^PCA+RBFNN model outperforms the PCA+RBFNN model and ICA+RBFNN models. From Table 3, almost all the measure indicators of the (2D)^2^PCA model are superior to those of the PCA and ICA models. In the first group experiment especially, the (2D)^2^PCA model shows much better performance than the other models. For example, the indicators PCD, *R* ^2^ and HR of the (2D)^2^PCA model reach 0.75309, 0.99369 and 0.73251, respectively, which are much larger than those of the PCA and ICA models. From the indicators, only the PCD of the (2D)^2^PCA+RBFNN model is less than that of the ICA+RBFNN model when the dimension is 50, and the other 8 indicators are superior to the ICA+RBFNN model. Comparing the (2D)^2^PCA+BPNN model with the PCA+BPNN model and ICA+BPNN model, the (2D)^2^PCA+BPNN model shows better performance than the other two models, and the RMSE 29.215 of the (2D)^2^PCA+BPNN model is smaller than that of the PCA+BPNN model (35.819) and ICA+BPNN (80.51).

Comparing the (2D)^2^PCA+BPNN model with the PCA+BPNN and ICA+BPNN models, when the input data are the same dimension, the former has better performance than the latter. Comparing the (2D)^2^PCA+BPNN model with the raw data+RBFNN model, the (2D)^2^PCA+BPNN model shows better performance than the raw data+RBFNN when the feature dimensionality is 11×11 = 121. This indicates that dimension reduction play an important role in accuracy of the predictive model. (2D)^2^PCA removes the redundant information from the raw data to improve the predictive accuracy. When the dimensionality is 7×7 = 49, not all measure indicators are better than that of raw data+RBFNN. The reason is that the dimensionality of the features is too small to extract enough useful information from the raw data.

From Table 3, we can see that the (2D)^2^PCA+RBFNN model obtains the highest total return, 1317.7, in this stock market set, with the dimension being 121. (2D)^2^PCA+RBFNN performs better than other models including raw data+RBFNN, whose return is 948.3. Another key issue of note is that the hit rate is not fully consistent with the returns from the figures in Table 3. For example, the hit rate of (2D)^2^PCA+RBFNN is higher than that of PCA+BPNN and ICA+BPNN in group 3. However, the returns of the former are lower than that of the latter. The reason for this is that hit rate only represents the frequency of the forecasting accuracy but does not take into account the fluctuation level of the stock market [14]. So when the actual price of the stock fluctuates drastically, both hit rate and the return should be considered to evaluate the performance of the forecasting model.

Table 4 shows the running time of the proposed model. In the four group experiments, the dimension reduction time of the (2D)^2^PCA models is much less than that of the PCA models; for instance, the (2D)^2^PCA model needs 2.687s while the PCA model requires 57.219s and the ICA model 84.326s in the first group experiment. Because the dimension of the (2D)^2^PCA model is quite close to the PCA model and ICA model, the RBFNN training time and the simulation time are close for the three types of model. It is also found that, in the four group experiments, because the convergence speed of the (2D)^2^PCA model is much faster than the other two models, the feature extraction time of the former is significantly less than the latter. In regard to total running time, due to the contribution of dimension reduction time, the (2D)^2^PCA model is also more powerful than the PCA model. Another phenomenon should be noted, namely that the (2D)^2^PCA+RBFNN model is more efficient than the (2D)^2^PCA+BPNN model from the point of view of the network training time in the fourth group experiment. The training time of (2D)^2^PCA+BPNN model is 14.891s which is 6.344s faster than that of the (2D)^2^PCA+RBFNN model. In fact, this difference is for *DIM* = 7; when the *DIM* is 49 and 121, for (2D)^2^PCA+BPNN, the network training time is prohibitively long. From the table, we can also find that the training time and the testing time of raw data+RBFNN are much larger than for the other RBFNN models because of the larger feature dimensionality. Comparing the (2D)^2^PCA+BPNN model with the raw data+RBFNN model, although the former requires feature extraction, due to the smaller dimensionality of the features, the training time, testing time and total time of the former method are much less than that of the latter.

Based on the finding in Figs 5 and 6, Tables 3 and 4, it can be found that the proposed (2D)^2^PCA+RBFNN model can produce the lower prediction error and higher return under the Shanghai stock market dataset. Therefore, the proposed model indeed results better prediction performance than PCA+RBFNN, ICA+RBFNN, PCA+BPNN and ICA+BPNN models.

## Conclusion

This investigation evaluates 36 technical variables for forecasting stock market short-term trends, and utilizes (2D)^2^PCA to reduce the dimension of the input data; RBFNN is combined with (2D)^2^PCA to build a forecasting model. The proposed approach with RBFNN models provides strong robust and adaptive capability in predicting the daily closing price, so it was able to cope with the fluctuation of stock market values and yielded good prediction accuracy. In addition, with fewer input variables generated by (2D)^2^PCA feature selection approach, the (2D)^2^PCA+RBFNN model can shorten the training time. To evaluate the efficacy of the proposed model, the (2D)^2^PCA+RBFNN model is applied to forecast the Shanghai stock market index and the results show that the proposed model has good performance. We compared the obtained forecasting accuracy (using multiple performance criteria) with those of the traditional model PCA+RBFNN. Additionally, to evaluate the performance of the RBFNN with that of a popular alternative regression model, we also compared with a (2D)^2^PCA+BPNN model and a PCA+BPNN model. The comparison shows that the forecasting ability of the (2D)^2^PCA+RBFNN model is better than the other models. Furthermore, due to the low complexity of (2D)^2^PCA for dimension reduction and the high convergence speed of the associated regression model learning, the proposed model shows better computational efficiency in stock market forecasting than its alternatives.

Overall, the results presented in this study have confirmed that the proposed model provides a promising method for stock forecasting. Although the proposed model provides many advantages, it also has minor weakness. While the model obtains high accuracy forecasting at low computational cost, the input dimension of the RBFNN is still high. In our experiments, the highest dimension was 121, and despite the fact that the RBFNN training has fast convergence, this high dimensionality and associated training complexity may not be suitable for some real-time forecasting contexts where models must be rapidly built, “on-the-fly”. To alleviate this problem, a possible way is to select more efficient technical variables. However, this is an open problem.

## Supporting Information

S1 Data Data of experiment. (XLS)
